# Supplementary material for: Maintenance and transformation of representational formats during working memory prioritization
Source: Nat Commun. 2024 Sep 19;15:8234. doi: 10.1038/s41467-024-52541-w (PMC11412997; doi:10.1038/s41467-024-52541-w)
Supplement: Supplementary file 1 — Supplementary Information [file 41467_2024_52541_MOESM1_ESM.pdf]

Supplementary Information

Maintenance and transformation of representational formats during working memory prioritization

Pacheco Estefan et al., *Nature Communications*, 2024.

Supplementary Figures 1-13

Supplementary Notes 1-7

Supplementary References

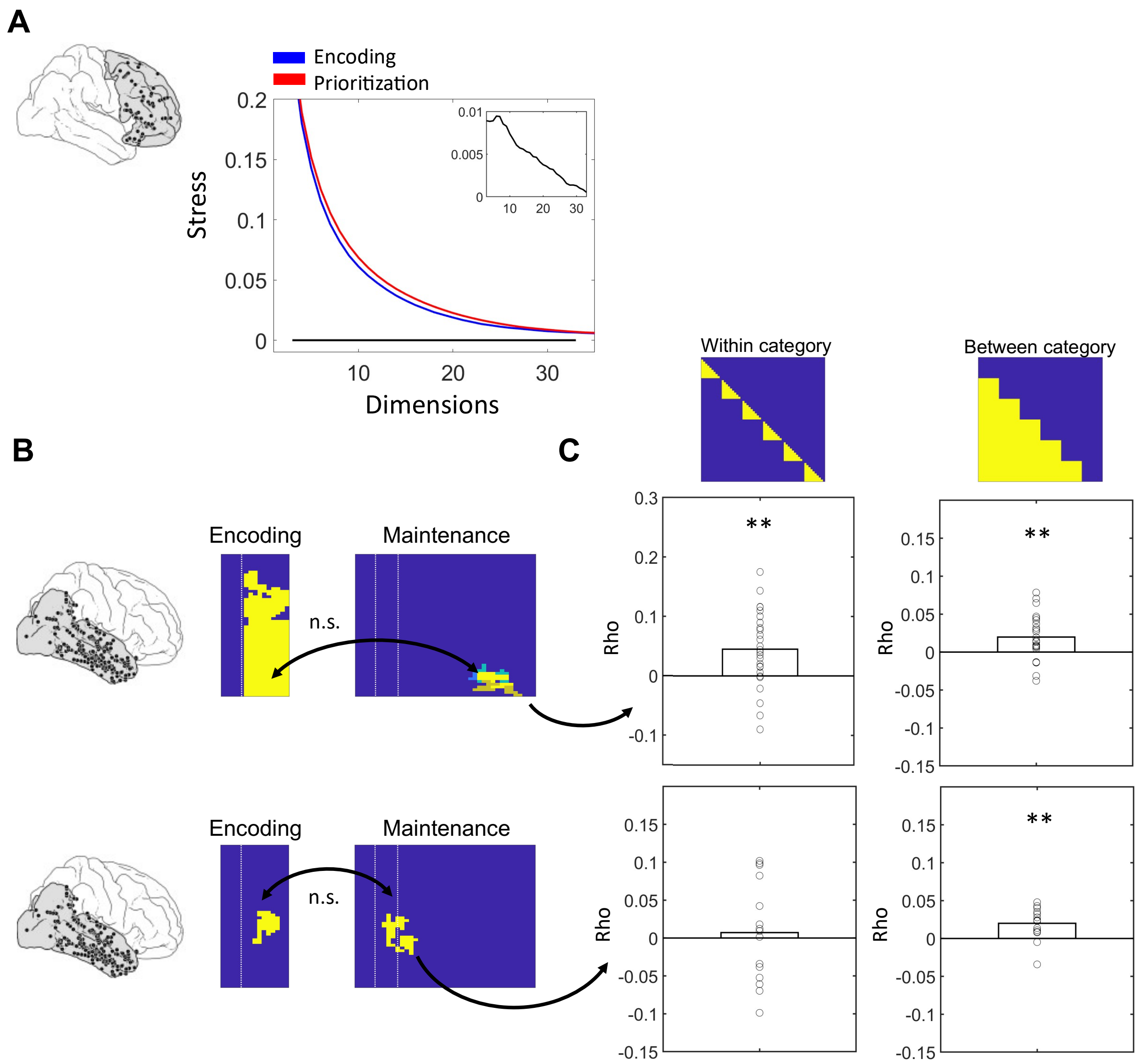

Supplementary Figure 1: Representational transformations during encoding and prioritization in VVS and PFC

(A) Group averaged stress values as a function of dimensionality during encoding (red) and prioritization (blue) in the PFC. Black line shows cluster of dimensions in which stress was significantly higher during maintenance as compared to encoding (corrected for multiple comparisons using cluster-based permutation statistics). Inset figure shows stress differences (prioritization – encoding) in the time period where significant differences were observed (dimensions 4-33). **(B)** Time-frequency regions where original fits with the category model during

encoding, and with BL-NET during maintenance were identified in VVS (top row) and the PFC (bottom row). Selected clusters in each region and time period are indicated in yellow. During the maintenance period in the VVS analysis, an intersection cluster (marked in yellow) was employed, based on the BL-NET analysis in layers 4 (blue), 5 (cyan) and 6 (brown). The fits in the two regions of interest were not correlated, indicating representational transformations in both regions (indicated with curved black arrows in the figure). **(C)** Left and middle column: Fit of BL-NET to the average RSMs extracted from the clusters depicted in panel A during the maintenance period for exemplars within categories (left) and between categories (right). In the within-category analysis, we observed that BL-NET representations matched neural representations only in the VVS ($t(26)= 3.85$, $p = 0.00071$), but not in PFC ($t(15)= 0.42$, $p = 0.67$). In the between-category analysis, the BL-NET significantly matched neural RSMs in both regions (PFC: $t(15) = 3.96$, $p = 0.0024$; VVS = $t(26) = 3.48$, $p = 0.001$).

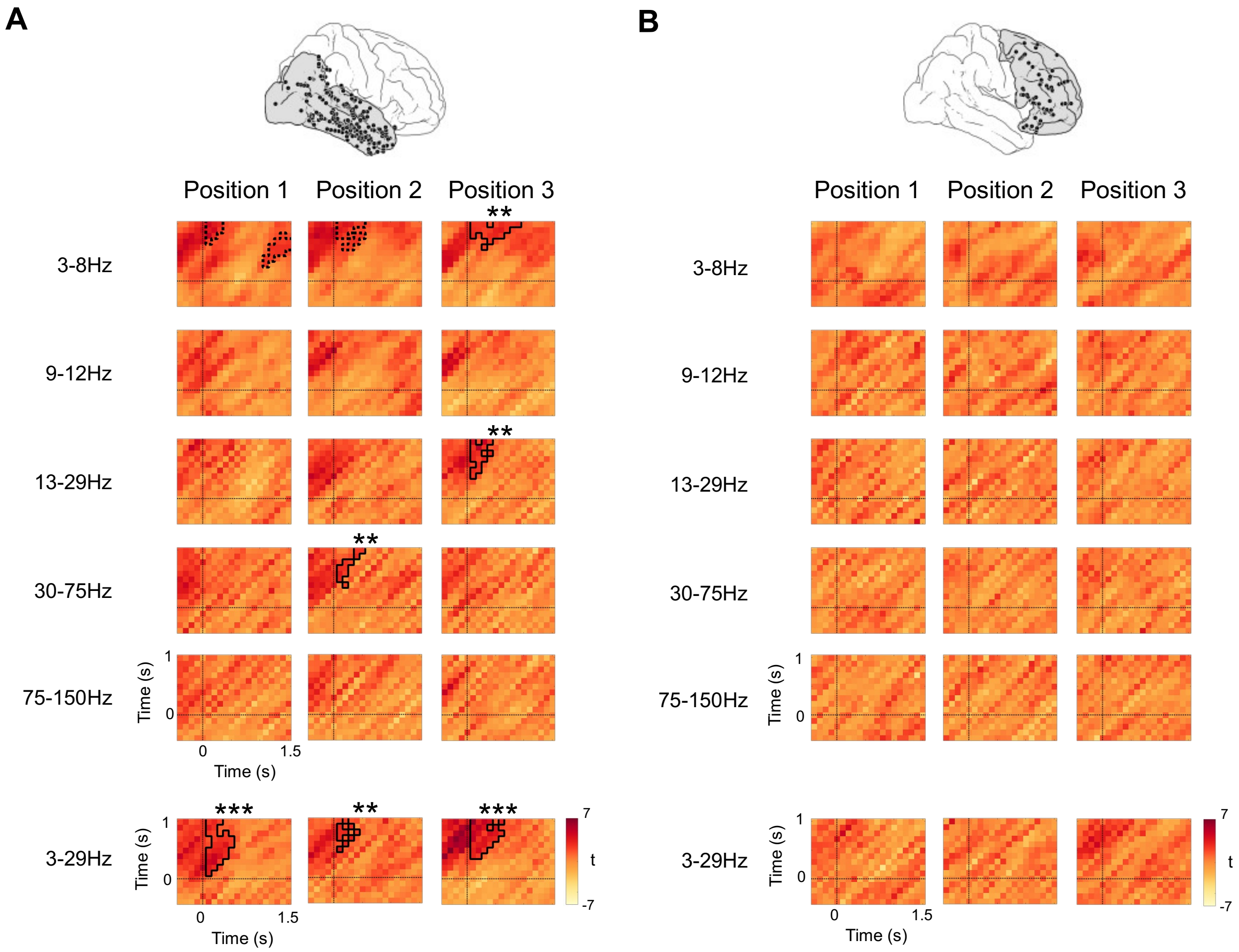

Supplementary Figure 2: Encoding-maintenance 1 similarity analysis

Encoding-maintenance similarity during the first maintenance period (EM_1S) was computed for each encoding position by correlating patterns of activity at different encoding time windows (vertical axis) with maintenance activity after the offset of the third image (horizontal axis), for the VVS **(A)** and the PFC **(B)**. This was done separately in five frequency bands (theta, alpha, beta, low gamma, high gamma), and in a broadband analysis including all low frequencies (theta, gamma, beta; bottom row in panels A and B). Clusters with significant differences between same and different categories surviving correction for multiple comparisons using cluster-based permutation statistics are outlined in black. Dashed lines in panel A indicate clusters at trend level ($p < 0.1$). Time zero in all panels indicates image onset during encoding and third image offset during maintenance, respectively. ***: $p < 0.001$; ** : $p < 0.01$.

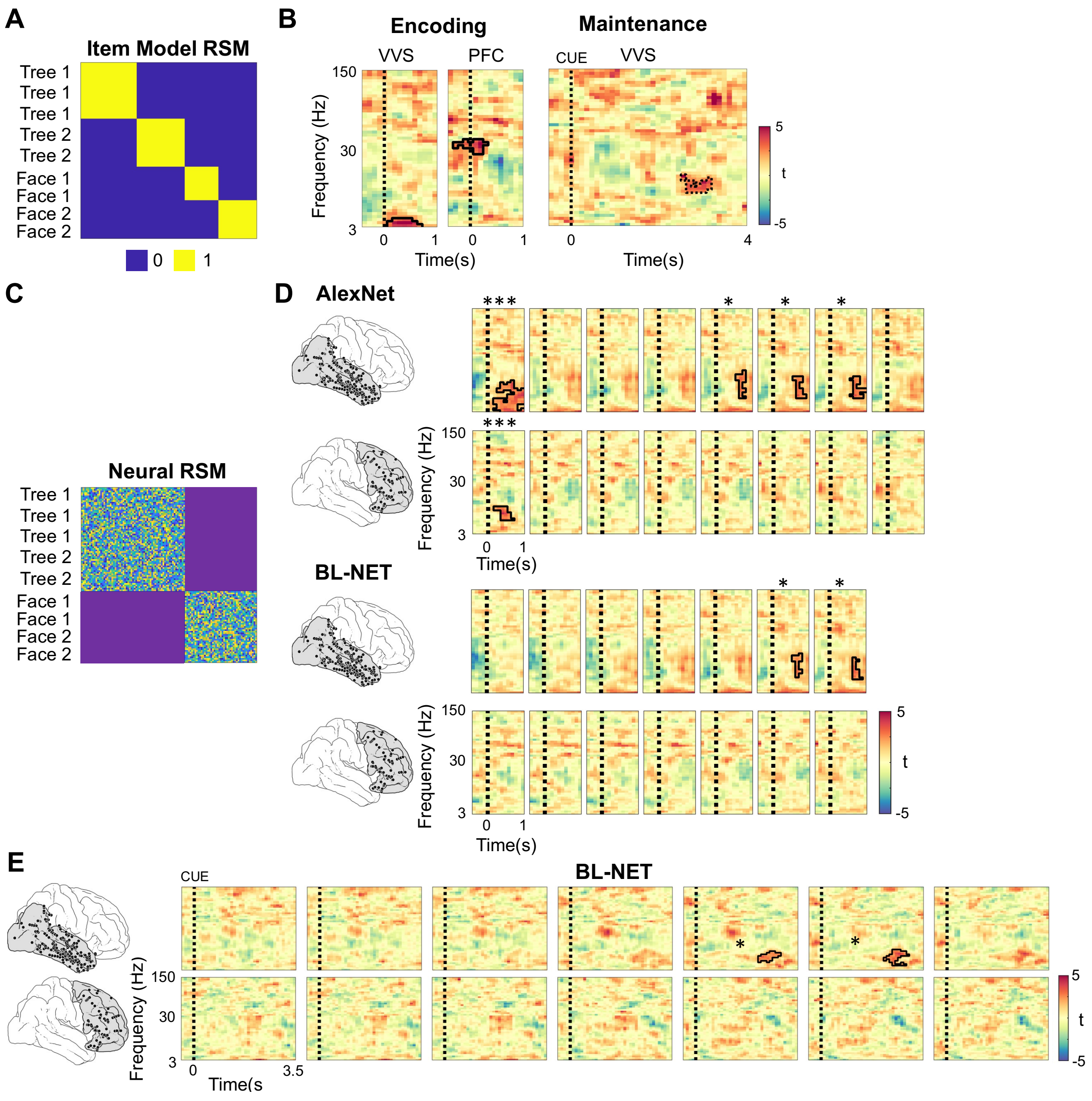

Supplementary Figure 3: Representation of individual exemplars

(A) The item model Representational similarity matrix (RSM) encodes the same exemplars with a 1 and different exemplars with a 0. Note that the repeated presentation of the same exemplars is required in this analysis. **(B)** Left: The item model significantly matched representations during encoding both in the VVS and the PFC. Regions outlined in black indicate significant periods after correction for multiple comparisons at $p < 0.05$ (cluster-based permutations statistics). Right: fit of the item model during maintenance in the VVS. Relatively high correlations were observed towards the end of the maintenance period in the alpha frequency range at a trend level ($p = 0.07$;

outlined with dashed lines in the plot). **(C)** Correlations between DNNs and neural RSMs were computed after excluding the between category correlations (depicted in purple in the example RSM). **(D)** The RSMs of the AlexNet and BL-NET networks significantly fit the neural data in the VVS in layers 1, and 4-6 (AlexNet) and layers 6 and 7 (BL-NET) in the theta and alpha-beta frequency ranges. In the PFC, a significant fit was only observed in the input layer of the AlexNet network in the alpha range. **(E)** During maintenance, the BL-NET network explained VVS representations towards the end of the maintenance period in the alpha frequency range, while no significant fit was observed in the PFC. ***: $p < 0.001$; * : $p < 0.05$.

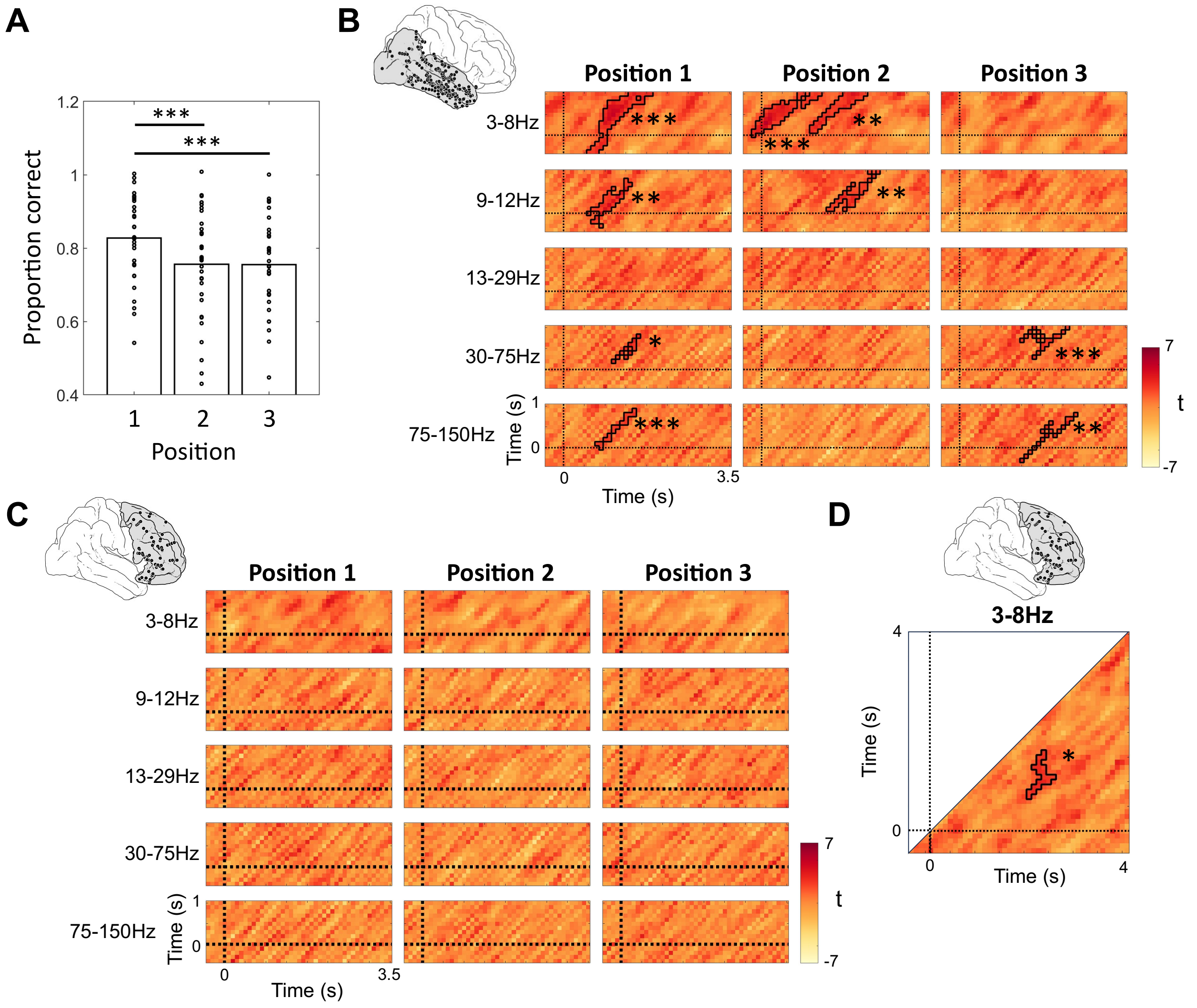

Supplementary Figure 4: Performance, EMS and MMS analysis for items encoded in positions 1-3

(A) Memory performance for items in positions 1, 2 and 3 collapsed across single and multi-item trials. **(B)** Category-specific EMS analysis was conducted independently for each position and frequency band in the VVS. Time zero in the x and y axes indicate onset of the retro-cue during maintenance and of stimulus presentation during encoding, respectively. **(C)** Same as in panel A for PFC data. **(D)** Within-category MMS similarity analysis in the theta (3-8Hz) frequency range shows significant differences between items encoded in the same versus different positions. Time zero in both axes indicates the onset of the cue. In panels B, C and D, clusters with significant differences between same and different positions surviving correction for multiple comparisons at $p < 0.05$ (cluster-based permutation statistics), are outlined in black. ***: $p < 0.001$; ** : $p < 0.01$; * : $p < 0.05$.

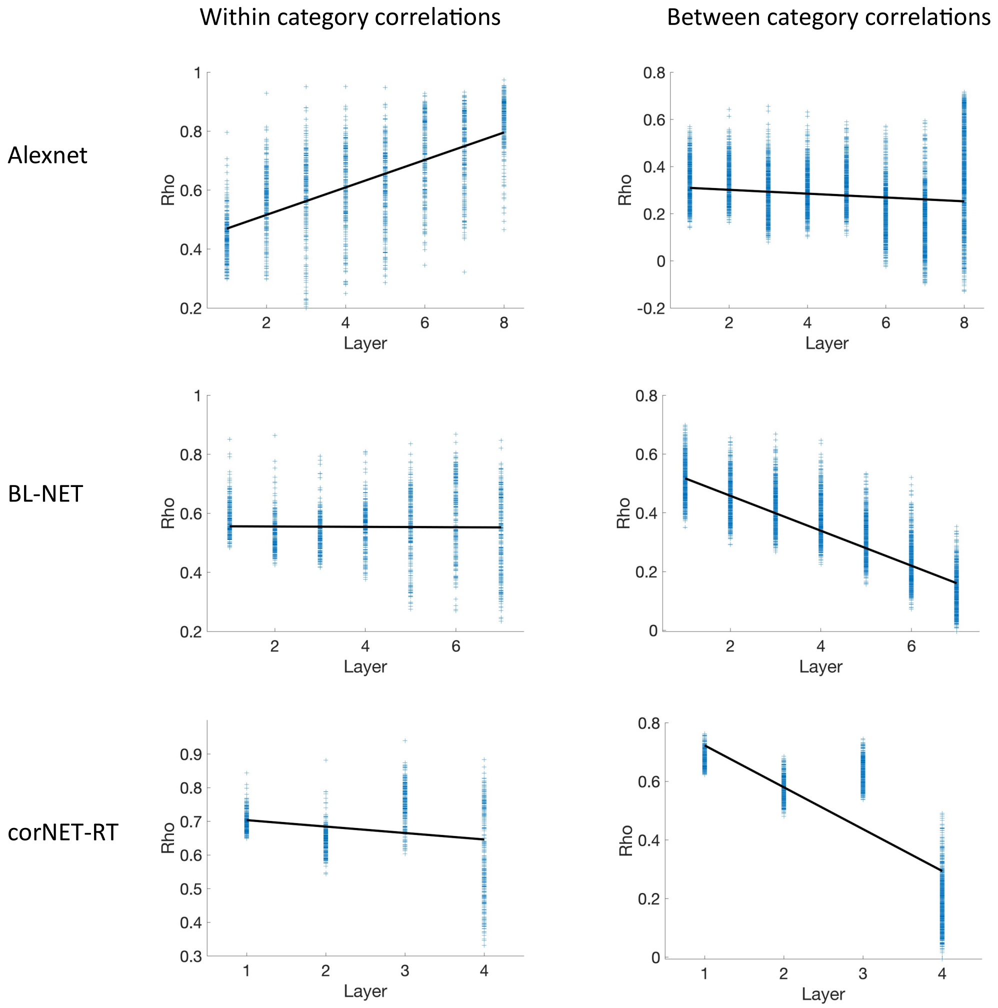

Supplementary Figure 5: Within-category and between-category correlations as a function of network layer for all employed DNNs

The figure shows all pairwise within-category (left column) and between-category (right column) correlations for AlexNet (top row), BL-NET (middle row), and corNET-RT (bottom row) networks. Black line in each plot shows linear fit performed over all pairwise comparisons in each network for visualization, but note that the statistics reported in the main text were computed by performing a linear fit to each of the individual item pairs separately, and contrasting the resulting distribution of slopes across networks and conditions.

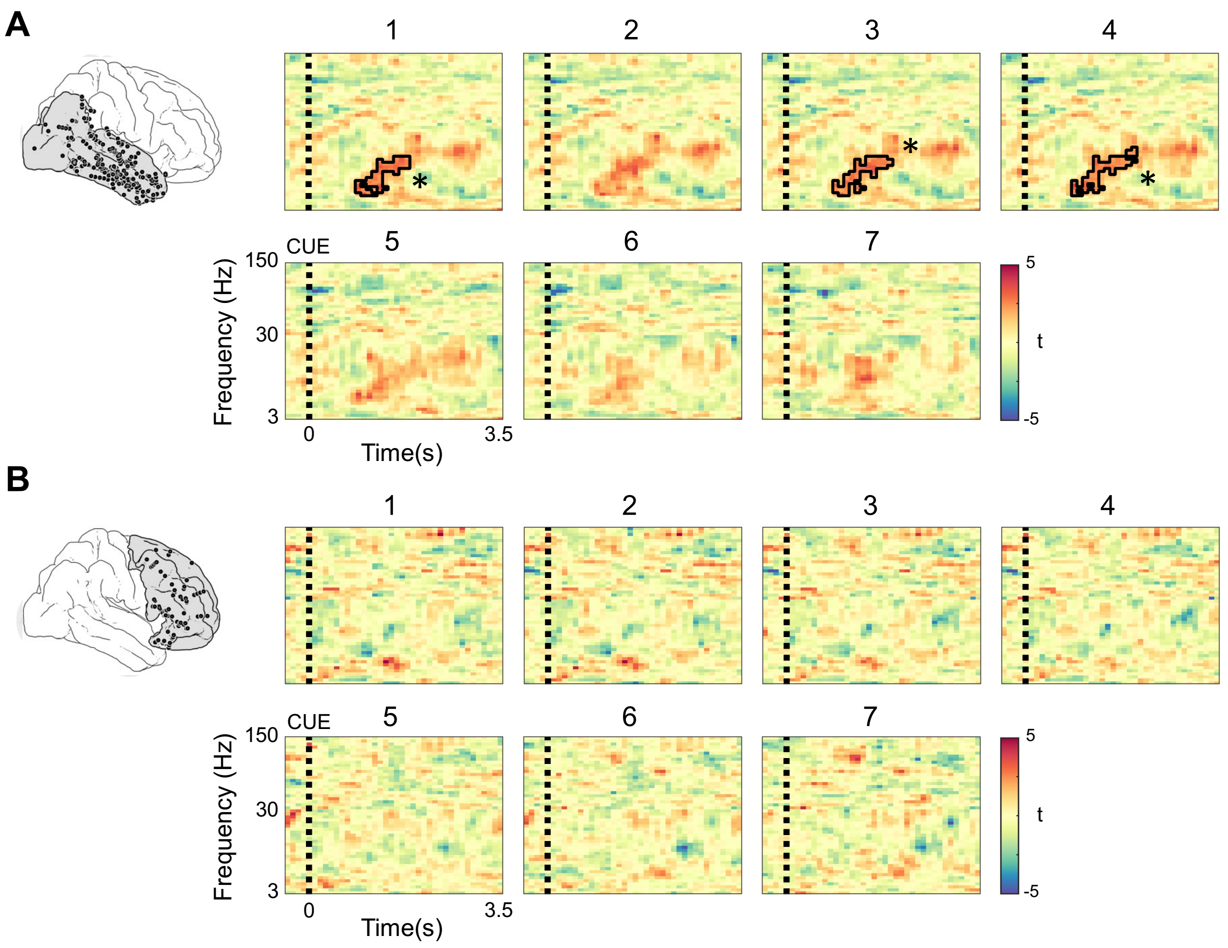

Supplementary Figure 6: Analysis of correct vs incorrect trials in BL-NET

To investigate the functional relevance of representations in VVS and PFC during the maintenance period, we contrasted levels of DNN fits for correct vs. incorrect trials. Results revealed significant differences between correct and incorrect trials in VVS but not in PFC. **(A)** In the VVS, fits were higher for correct as compared to incorrect trials in BL-NET layers 1 (p_{corr} = 0.014), 3 (p_{corr} = 0.028) and 4 (p_{corr} = 0.021; Bonferroni corrected for 7 network layers. This was observed in three clusters of significant time frequency bins in the alpha-beta range (6-21Hz), which started after the onset of the cue and lasted for ~1s in each of the three layers (outlined in black). Notably, these clusters were observed in the same time-frequency period where we observed significant EMS in the VVS (Figure 2). **(B)** In the PFC, no significant clusters were observed in any layer (all p_{corr} = 1). These results demonstrate that category-specific representations were substantially degraded in the incorrect trials as compared to the correct trials in the VVS during the maintenance period. Notably, these effects occurred in a time period which partially overlaps with the significant EMS observed in this region. In the PFC, the

prioritization process appears to affect both correct and incorrect trials, since no difference between these two types of trials was observed in this region. In panels A and B, significant time-frequency regions after correction for multiple comparisons at $p < 0.05$ are outlined in black. * : $p < 0.05$.

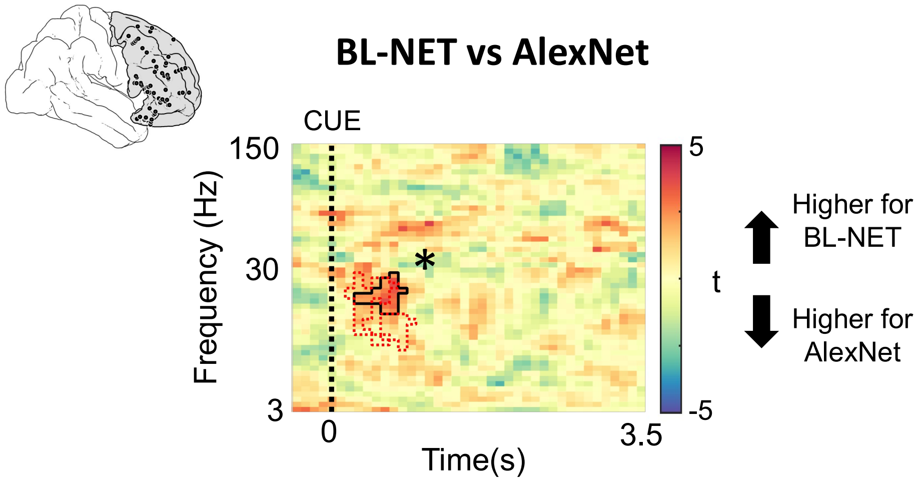

Supplementary Figure 7: BL-NET vs AlexNet model fits.

Figure shows the contrast of the AlexNet and BL-NET fits in the PFC in their corresponding deepest layers (BL-NET: layer 7; AlexNet: Fc8) during the maintenance period. A cluster of significantly higher correlations for BL-NET was observed in the 20-27Hz frequency range (p_{corr} = 0.01; outlined in solid black lines), overlapping with the cluster observed in the original BL-NET analysis (red dashed lines). Cluster-based permutation statistics were applied to correct for multiple comparisons in the restricted time period of item prioritization (0-1s after cue onset). Time zero indicates the onset of the cue during maintenance. * : $p < 0.05$.

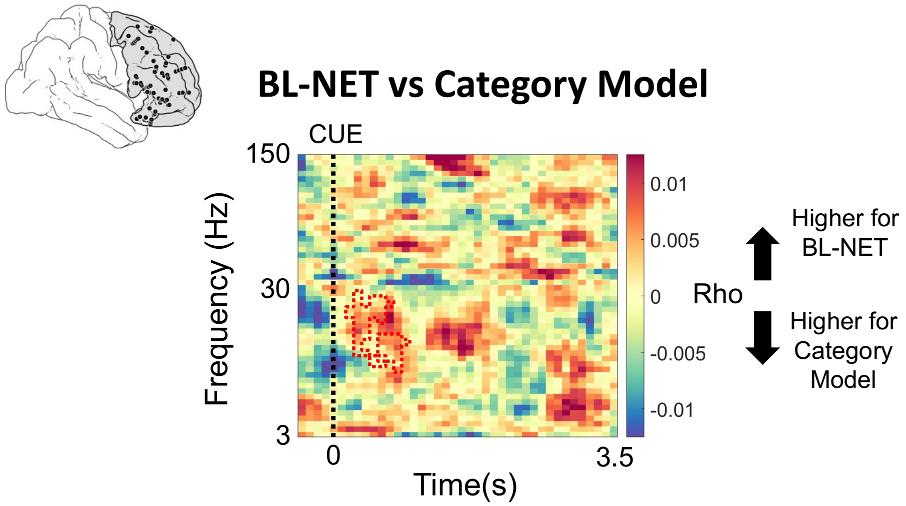

Supplementary Figure 8: Comparison of BL-NET and Category model Fits

Difference in BL-NET and category model fits in the PFC. Cluster outlined in red dashed lines indicates time-frequency regions where significant fits of BL-NET were observed in PFC.

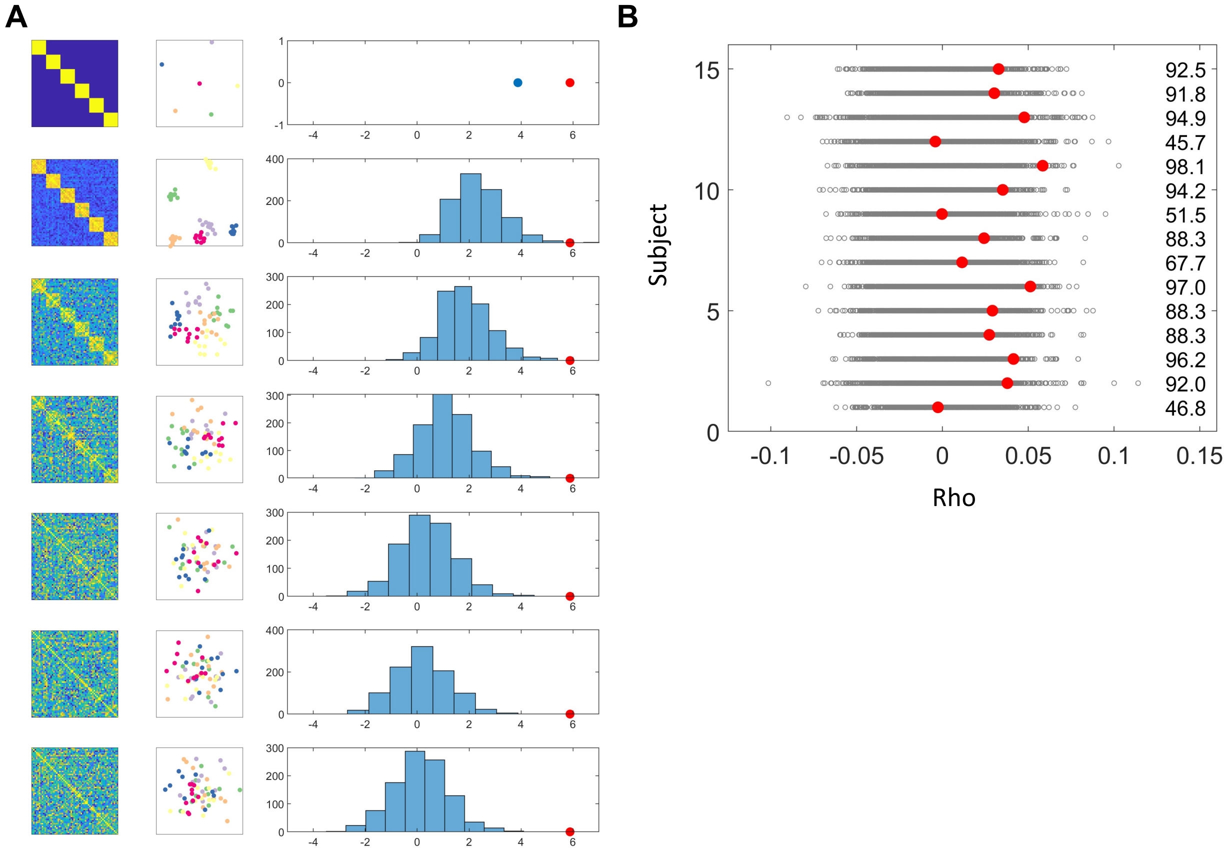

Supplementary Figure 9: Simulation analysis under different levels of noise and subject specific BL-NET fits to PFC

(A) Normally distributed random numbers with a mean of 0 and standard deviations of 0.1, 0.5, 1, 3, 5 and 10 were added to the category model RSM. The resulting RSMs (left column) together with their correspondent MDS projections (middle column) are shown. Right: Correlations between category model and PFC RSMs under different levels of noise. Note that the first row corresponds to the category model without added noise, and therefore a single value indicating to the correlation of the category model and BL-NET RSMs is shown (blue dot). In rows 2-7, distributions for each level of noise are included (number of samples = 1000). Red circle shows the correlation of PFC activity with BL-NET. **(B)** Subject-level correlation of BL-NET and PFC representational geometries. Null distributions of BL-NET fits to PFC were generated for every subject by randomly shuffling the identity of the network image labels. Observed correlations were higher than the mean null distribution in all subjects except two (subjects 1 and 12). Values on the right indicate the rank of the observed correlation in the null distribution.

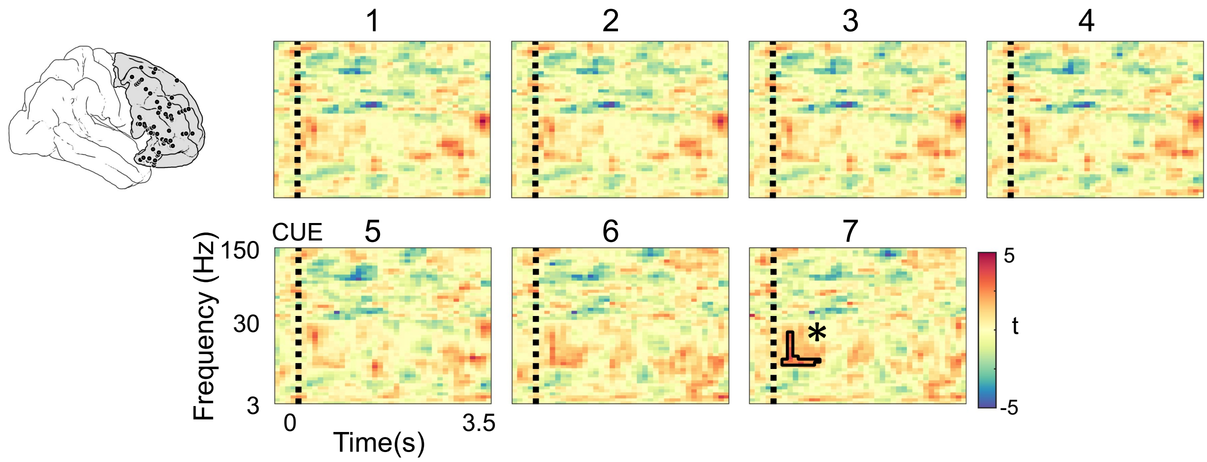

Supplementary Figure 10: Ecoset trained BL-NET

To verify that our results were not driven by the particular dataset with which the BL-NET was trained (i.e., ImageNet, as in previous studies, see refs. [1–10]), we performed the DNN model fit analyses with a variant of this network trained with a recently released dataset of images [11]. The 'Ecoset' dataset includes >1.5 million images from 565 categories which were selected to capture the distribution of objects relevant to humans in natural conditions, thus providing an ecologically valid alternative to ImageNet. We computed BL-NET fits for the PFC during the maintenance period employing the Ecoset-trained network weights. A significant matching between the representational geometry of the Ecoset-trained BL-NET network (layer 7) and that of PFC activity was found ($p = 0.016$), while the other layers did not show significant fits. * : $p < 0.05$.

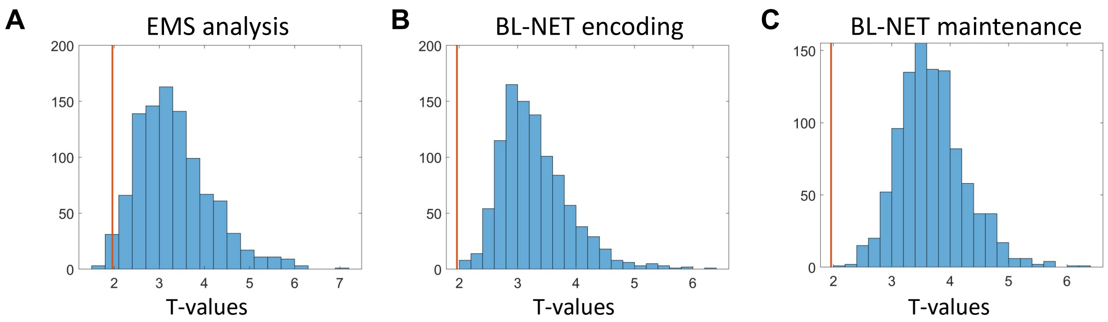

Supplementary Figure 11: EMS and DNN model-based analyses with matched numbers of participants in VVS

(A) EMS analysis was performed by randomly subsampling subjects in the VVS to match the number of subjects in the PFC (N=15). Histogram shows the counts of the resulting t-values of the group level correlations against zero (averaged during the whole encoding time period, and between 0-1.5s after cue offset during maintenance). Group level correlations were significant in 95.8% of subsamples. **(B)** The analysis of BL-NET fits to VVS data at encoding (layer 6) shows that a 100% of the tests performed by randomly subsampling subjects to match the number of subjects in PFC (N= 15) was significant. **(C)** The BL-NET analysis during maintenance (layer 6) shows that a 100% of the tests performed by randomly subsampling subjects in VVS to match the number of subjects in PFC (N= 15) was significant. Red line in all plots indicates the significance threshold of the t-distribution (p = 0.05).

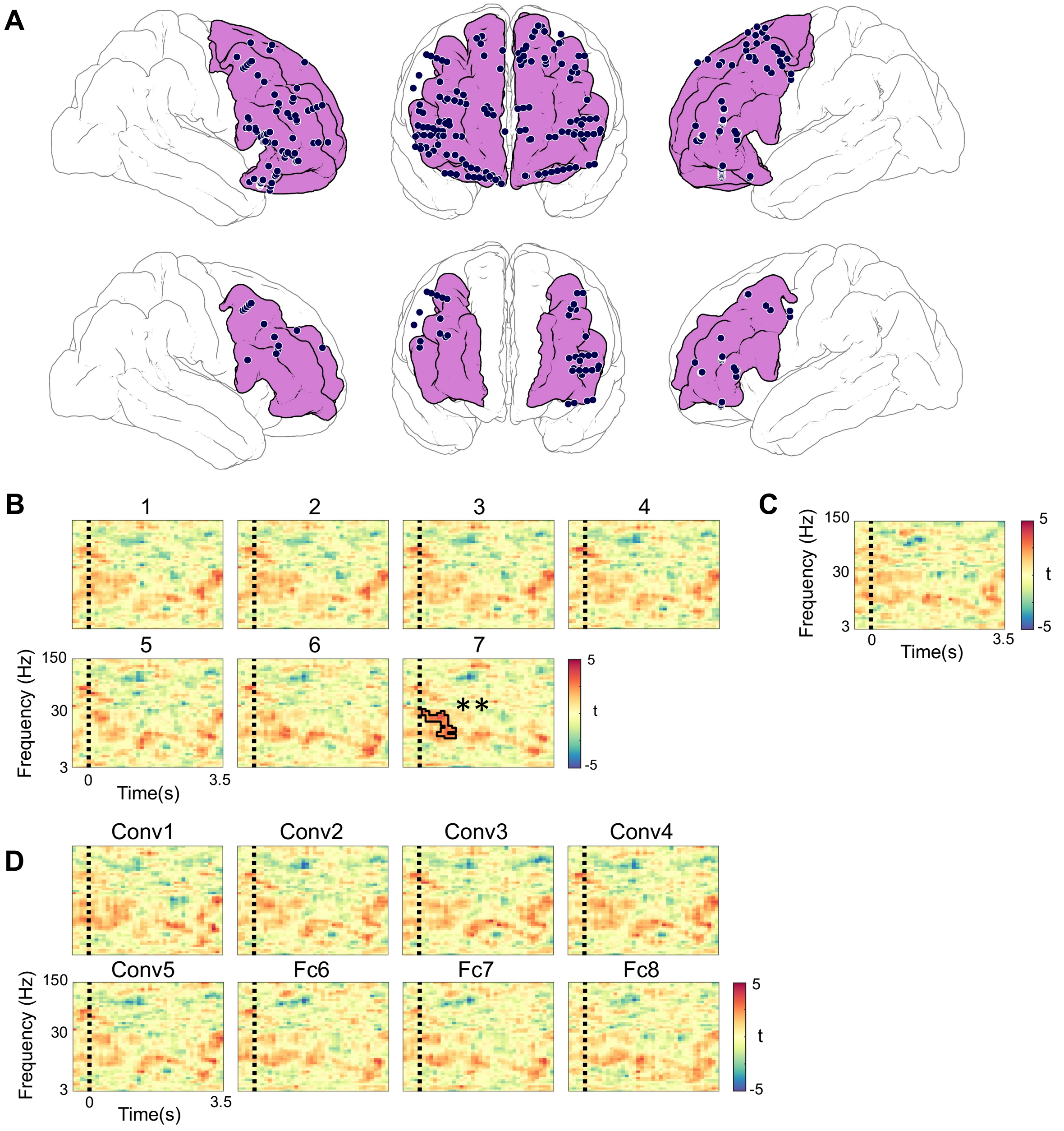

Supplementary Figure 12: BL-NET, Category Model and AlexNet fits in the lateral prefrontal cortex

Previous studies have underscored the relevance of the lateral prefrontal cortex (LPFC) in attentional prioritization [12], the representation of rules [13] and categories [14]. We thus performed all the model-based analysis in the PFC on a subset of electrodes located in this region during the maintenance period, after the presentation of the retro-cue. Only electrodes with MNI x-coordinates larger than -35 or smaller than +35 and z-coordinates larger than -15 were included in the LPFC analysis **(A)** From the 157 electrodes in 15 subjects included in the original analysis

(top row), a total of 9 subjects had at least one electrode LPFC (both hemispheres included). Bottom row: a total number of 38 channels were included in the LPFC analysis. Freesurfer regions corresponding to the areas where electrodes are located in each analysis are highlighted in pink. Panels B, C and D show the fit of different models to the representational geometry of stimuli in LPFC during the maintenance period: **(B)** BL-NET, **(C)** Category model, **(D)** AlexNet. Time 0 (dashed line) indicates the onset of the retro-cue and the start of the maintenance period in all plots. A fit was selectively observed in the final layer of BL-NET during the prioritization period (panel B; $p_{corr} = 0.007$, Bonferroni corrected for 7 layers), while no significant fits were found in any other layers (all $p_{corr} > 0.33$). Consistent with our original analyses, we did not observe any significant fit in the analysis of the category model (panel C; all $p > 0.47$), and with any layer of the AlexNet network (Panel D; all $p_{corr} > 0.476$). Regions outlined in black show significant time-frequency bins after correction for multiple comparisons at $p < 0.05$ (cluster-based permutation statistics). * : $p < 0.05$.

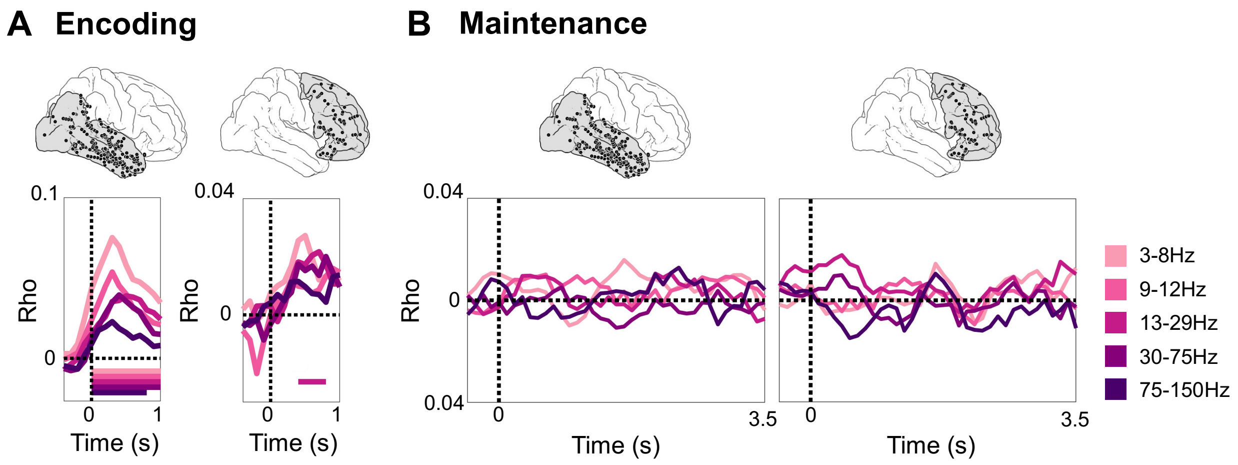

Supplementary Figure 13: Category model fits for 5 frequency bands

(A) Category model fits during encoding for VVS (left) and PFC (right) for five frequency bands color coded from pink (theta) to dark purple (high gamma). Each line shows the rho values of a particular frequency band. Horizontal lines at the bottom of each plot indicate time periods where rho values are significantly higher than zero at the group level (Bonferroni corrected for five bands). **(B)** Category model fits during maintenance for VVS (left) and PFC (right).

Supplementary Note 1: Analysis of representations during the maintenance 1 period

We computed the fit of all DNNs during the first maintenance period (before the presentation of the cue, 'M1' in Figure 1A). Since there is no 'cued' item during this period, and to assess a possible sequential representation of each item, we computed the fits separately for each of the presented items (position 1, 2 and 3). These analyses did not reveal a significant fit during the M1 period for any of the items presented in the sequence (position 1: all $p_{corr} > 0.77$; position 2: all $p > 0.2$; position 3: all $p_{corr} > 0.51$).

Additionally, we performed a maintenance-maintenance similarity analysis (MMS) in which we calculated M1-M1 similarity between trials in which all items that were presented during encoding matched, versus trials in which all items are different. Results in this analysis revealed a lack of significant fits in all five frequency bands we tested (VVS: all $p_{corr} > 0.47$; PFC: all $p_{corr} > 0.71$, Bonferroni corrected for five frequency bands). While this analysis is sensitive to a "compound" representation of all presented items, it is not well suited to detect a possible sequential representation of the items.

Finally, consistent with our original approach, we also computed an encoding-maintenance similarity analysis in the M1 period (EM_1S analysis) for five frequency bands (Theta, Alpha, Beta, Low Gamma, High Gamma). We computed EM_1S separately for items at the first, second and third position, to assess a possible relevance of the sequential order of representation of the items. In the VVS, we observed significant EM_1S for items encoded at the third position in the theta range ($p_{corr} = 0.025$, Bonferroni corrected for 5 bands); for items at the first and the second position, this effect only reached significance before Bonferroni correction (first position, $p = 0.028$; second position: $p = 0.027$). Similarly, we found that items encoded at the third position showed significant EM_1S in the beta range ($p_{corr} = 0.005$), while we observed EM_1S at a trend level in position 2 ($p_{corr} = 0.06$), and no effects in position 1 ($p_{corr} = 0.134$). We also observed significant EM_1S in the gamma band for items in position 2 ($p_{corr} = 0.015$), but not in position 1 or 3, which did not reach significance at an uncorrected level ($p > 0.11$). Following up on these results, we performed an analysis in which we grouped together the low frequencies (theta, alpha and beta; 3-29Hz) and assessed EM_1S in the VVS. Results revealed marked EM_1S in this broad frequency range in the VVS for items encoded in all positions (position 1: $p_{corr} = 0.005$; position 2: $p_{corr} = 0.015$; position 3: $p_{corr} = 0.005$), confirming that representations of encoded items are maintained in the VVS during the M1 period, before the presentation of the cue. Our EM_1S analysis in the PFC did not show any significant result in any of the five frequency bands (all $p_{corr} > 0.53$).

Supplementary note 2: Representation of individual exemplars

To evaluate the presence of item-specific information in our data, we built a model RSM which encodes the same exemplars with a 1 and different exemplars with a 0 (Supplementary Figure 3A). Note that for this analysis, multiple repetitions of the same item are required, and these repetitions are not averaged as in the main analysis. The item model was correlated with a time-series of neural RSMs at each individual frequency. During encoding, the item model significantly matched the neural RSMs in the theta (3-5 Hz; $p = 0.009$) frequency range in the VVS, and in the beta frequency range (25-29Hz) in the PFC ($p = 0.02$). During maintenance, a trend was observed in the VVS towards the end of the maintenance period, which did not survive corrections for multiple comparisons ($p = 0.07$). These results are presented in Supplementary Figure 3B.

In addition, we evaluated whether the DNNs could distinguish individual exemplars within categories. We computed correlations between neural RSMs and model RSMs at all time-frequency points in VVS and PFC after excluding the between-category correlations (Supplementary Figure 3C). In the VVS, we observed significant fits with item-level AlexNet representations of layer 1 ($p_{corr} = 0.008$), layer 5 ($p_{corr} = 0.04$), layer 6 ($p_{corr} = 0.016$) and layer 7 ($p_{corr} = 0.024$) and with item-level BL-NET representations of layer 6 ($p_{corr} = 0.028$) and layer 7 ($p_{corr} = 0.007$). Notably, the presence of item-specific information was not only observed in superficial layers in our architectures, but also in intermediate and deep layers, which underscores the relevance of high-level visual information for the representation of individual exemplars. In the PFC, we observed a significant fit with AlexNet representations in layer 1 during encoding ($p_{corr} = 0.024$), while no significant fits with BL-NET representations were observed (all $p_{corr} > 0.28$, Supplementary Figure 3D). During maintenance, we focused solely on the BL-NET network because AlexNet did not show any significant fits in the original analysis. In the VVS, results revealed significant matching with exemplar-specific BL-NET representations in layers 5 and 6 in a time-frequency period that overlapped with the original fits observed in the main analysis (layer 5: $p_{corr} = 0.014$; layer 6: $p_{corr} = 0.014$, Bonferroni corrected for seven layers, Supplementary Figure 3E). No significant clusters were observed in the PFC (all $p_{corr} = 1$, Supplementary Figure 3E). Taken together, these findings reveal that the representational transformations in both regions resulted in a dissociation of the coding scheme of VVS and PFC in terms of their representational content, with a more prominent reliance on within-category correlations in the VVS than the PFC.

Supplementary Note 3: Modulation of behavior and neural representations by position of the items at encoding

We evaluated whether behavioral performance for items at different positions differed in our task. We constructed a 2x2 repeated measures ANOVA to test the effect of position and type of trial (single or multi-item) on the overall memory performance. Performance at each position was computed as the number of correct responses divided by the total number of responses. Results of the ANOVA model revealed that indeed both position and type of trial had a significant effect on memory performance (position: $F(31) = 18.52$, $p = 4.72e-07$, type of trial: $F(31) = 10.28$, $p = 0.0031$). Items encoded in the first position were remembered significantly better than items in the second position ($t(31) = 5.11$, $p_{Tukey} = 4.55e-05$), and in the third position ($t(31) = 4.65$, $p_{Tukey} = 0.0001$). No significant difference was observed between items in the second and the third position ($t(31) = 0.09$, $p_{Tukey} = 0.99$). Moreover, performance for the single item trials was significantly better than performance for the multi-item trials ($t(31) = 3.21$, $p_{Tukey} = 0.0031$). In addition to the main effects of position and type of trial, we also observed a significant interaction between these two factors ($F(31) = 4.515$, $p = 0.0148$). We therefore conducted two separate one-way ANOVAs to assess the effect of position separately for single and multi-item trials. In the single-item condition, we found a main effect of item position ($F(31) = 3.279$, $p = 0.044$). Post-hoc analyses revealed a trend for higher performance of items encoded in positions 1 vs 2 ($t(31) = 2.45$, $p_{Tukey} = 0.0509$), and no significant differences between positions 1 and 3 ($t(31) = 1.36$, $p_{Tukey} = 0.0509$), or 2 and 3 ($t(31) = -1.26$, $p_{Tukey} = 0.22$). In the multi-item trials, we also found a main effect of item position ($F(31) = 38.23$, $p = 1.52e-11$), and post-hoc analyses revealed higher performance for items encoded in positions 1 vs 2 and 1 vs 3 (1 vs 2: $t(31) = 6.06$, $p_{Tukey} = 3.0e-06$; 1 vs 3: $t(31) = 7.24$, $p_{Tukey} = 1.157e-07$), and higher performance for position 2 vs 3 ($t(31) = 2.93$, $p_{Tukey} = 0.017$). Thus, we observed primacy but no recency effects.

Given that the positions in which the items were encoded affected their probability of recall, we investigated whether the neural representations were also affected. First, to assess the presence of ‘rule’ representations, we evaluated whether items encoded in the same position had greater similarity than items encoded in different positions during the maintenance period (maintenance-maintenance similarity analysis, MMS). In this analysis, the original position of the cued item was taken, and contrasts were conducted separately for items of the same category (excluding same-exemplar correlations) and for items of different categories. We used the same temporal resolution as in the original analysis (i.e., 500ms windows sliding in 100ms; Figure 2D).

Second, we assessed the reinstatement of category-specific similarity for items encoded at different positions (Encoding-maintenance similarity analysis, EMS).

(A) MMS analysis: Representation of position information in PFC but not VVS

During maintenance, the within-category analysis revealed that position information was significantly encoded in PFC representations in the theta frequency range ($p_{corr} = 0.02$; Bonferroni corrected for 5 bands; Supplementary Figure 4D). This was observed in a time period from 700ms to 1.6s after the presentation of the cue. The other frequency bands did not show a significant effect, even at an uncorrected level (all $p > 0.289$). We did not find any significant effect of item position in any band in the between-category correlations analysis (all $p_{corr} > 0.52$). In the VVS, none of the analyses revealed a significant effect of position (within-category: all $p > 0.362$; between-category: all $p_{corr} > 0.3$). Notably, these results show that during the maintenance period, the PFC encodes information related to the position of the items, while this information is not present in the VVS.

(B) EMS analysis: Reinstatement of category-specific information from items at individual positions in VVS but not PFC

To evaluate whether category-specific information was more prominent for items encoded at particular positions, we performed EMS analyses contrasting within-category vs. between-category correlations, separately for items encoded in positions 1, 2, and 3. The EMS analysis revealed a similar pattern of results as the main analysis in the VVS and in the PFC (Supplementary Figure 4B and 4C). In the VVS, we found significant EMS for position 1 in all bands except for the beta band (all $p_{corr} < 0.03$, beta: $p_{corr} = 0.2$) while position 2 showed significant effects in the theta and alpha bands (theta: $p_{corr} = 0.005$, alpha, $p_{corr} = 0.015$, other bands: all $p_{corr} > 0.44$; Supplementary Figure 4B). Position 3 showed significant EMS in the gamma ($p_{corr} = 0.02$) and high gamma bands ($p_{corr} = 0.01$), while the other bands did not show an effect (all $p_{corr} > 0.1$). In the PFC, we did not observe EMS in any of the positions and bands we tested (all $p_{corr} > 0.3$; Supplementary Figure 4C), consistent with the lack of EMS observed in the original analysis in this region (Figure 2).

(C) Coding of position information in PFC lacks category specificity

To evaluate whether the behavior of the subjects (better performance for items in the first position) was reflected at the levels of EMS at each position, we directly contrasted category-specific EMS using a one-way within-subjects ANOVA with the factor “Position”, essentially corresponding to

an interaction analysis of category and position information. We subtracted within-category and between-category correlations for each of the encoding positions and averaged these differences during the full encoding time period (0-800ms) and the prioritization time period (0-800ms after cue onset). Results revealed a lack of significant differences in levels of EMS for items encoded at different positions: No effect of position was observed in any frequency band in VVS (Encoding: all $F(25) < 1.48$; all $p_{corr} > 1$, Prioritization: all $F(25) > 4.27$; all $p_{corr} > 0.097$) or in PFC (Encoding: all $F(14) < 1.75$; all $p_{corr} > 0.95$; Prioritization: all $F(14) < 1.79$; all $p_{corr} > 0.92$). In the PFC, we also performed this interaction analysis in the frequency band and time period where we observed significant position MMS effects (3-8Hz, 700ms to 1.6s after the presentation of the cue). Results revealed no significant effect of position in EMS in this time period either ($F(14) = 0.231$, $p = 0.795$).

Taken together, these results demonstrate that the position at which the items were encoded significantly influenced PFC representations during maintenance. Notably, the effect of position was observed in an overlapping time period but a different frequency range (i.e., theta, 3-8Hz) as compared to the results observed in the DNN analyses (i.e., beta frequency range, 15-29Hz, 0.2-1s after the presentation of the retro-cue). These results thus suggest that PFC representations of different task variables rely on separable neural signatures. Importantly, we also note that EMS does not interact with position information in the PFC, suggesting that the lack of EMS in PFC is not due to the influence of a position code. Indeed, our results demonstrate that PFC representations encode stimulus features and task variables via separable neural signatures, embedding categorical, high-level visual and task representations in a multiplexed neural coding scheme.

Supplementary Note 4: BL-NET, AlexNet and Category model comparisons

We performed additional analyses to compare the predictive performance of BL-NET, AlexNet and the category model using an aggregate measure of model fits. In each analysis, we selected only one layer of our DNNs and one particular time-frequency period, and we averaged levels of fits in each model. We performed statistical comparisons separately for the PFC and the VVS, given the distinct temporal features our two ROIs showed during the maintenance period.

In the PFC, we selected the last layer of our DNNs and focused specifically on the prioritization period (0-800ms after cue onset during maintenance) in the high-beta frequency range (21-29Hz). We constructed an ANOVA model with "RSM model" as a factor and included the average fits during this period for each of our different model RSMs. Results revealed a main effect of RSM model, indicating significantly different matching to the different models ($F(14) = 4.9$, $p = 0.0149$). Post-hoc analyses revealed that this effect was mostly driven by differences in fits between BL-NET and AlexNet ($t(14) = 3.01$, $p_{corr} = 0.0238$), while no significant differences were observed between BL-NET and the category model ($t(14) = 0.91$, $p_{corr} = 0.64$), nor between AlexNet and the category model ($t(14) = -2.07$, $p_{corr} = 0.133$, Tukey-Kramer corrected for three comparisons). We note that we considered a prioritization time period of the same length of that of stimulus presentation during encoding (i.e., 800ms after cue onset), but we observed similar results in the more restricted period of cue presentation (0-500ms; Main effect: $F(14)= 4.05$, $p = 0.029$, BL-NET vs. AlexNet = $t(14) = 2.66$, $p_{corr} = 0.045$; BL-NET vs. category model: $t(14) = 0.78$, $p_{corr} = 0.72$; AlexNet vs. category Model: $t(14) = -1.91$, $p_{corr} = 0.17$; Tukey-Kramer corrected for three comparisons).

In a complementary analysis with a more comprehensive and data-driven approach, we contrasted BL-NET and AlexNet fits during a more extended time period of item prioritization and across all frequencies (0-1s after cue onset during maintenance, 3-150Hz range). We used the same temporal resolution as in the original analyses, i.e., 100ms steps. Cluster-based permutation statistics were applied to correct for multiple comparisons by shuffling the condition labels (network identity). This analysis revealed that representations in the last layer of BL-NET provided significantly better fits than representations in the last layer of AlexNet in a cluster from 200 to 800ms after the onset of the cue in the 20-27Hz frequency range ($p_{corr} = 0.01$; Supplementary Figure 7). We performed the same analysis now contrasting BL-NET layer 7 with AlexNet layer Fc7 instead of Fc8. We chose the penultimate fully connected layer instead of the very last layer of AlexNet because this layer contains a relatively higher number of features, which increases variance and has been selected in other studies (e.g., ref. [9]). Again, we observed a

cluster of significant time-frequency bins from 400ms and until 800ms in the high beta range (21-26Hz, p_{corr} = 0.018).

In the VVS, we selected the end of the maintenance period (2-3s after cue onset) in the alpha frequency range (9-12Hz) and compared BL-NET (layer 5), AlexNet (layer 5), and the category model, again using an ANOVA. Results revealed a significant main effect of model in this time-frequency period, again indicating different degrees of matching to the 3 models ($F(25) = 6.65$, $p = 0.0027$). Post-hoc comparisons indicated that differences in the model fits were driven by differences between BL-NET and the category model ($t(25)= 2.71$, $p_{corr} = 0.03$) and between AlexNet and the category model ($t(25)= 2.65$, $p_{corr} = 0.035$), while the difference between BL-NET and AlexNet was not significant ($t(25) = -0.16$, $p_{corr} = 0.98$; Tukey-Kramer corrected for 3 comparisons). Notably, the lack of significant difference between the AlexNet and the BL-NET is consistent with the results reported above that VVS representations could also be fit by representations in the BF-NET, suggesting they rely less on recurrency than those in PFC.

Supplementary Note 5: Comparison of BL-NET and Category model fits under different levels of noise, and individual participants analysis

We quantified the similarity of the category model and the deep layer rDNN fits, focusing specifically on the BL-NET model (layer 7). We computed the difference in the two models' fits in every subject and contrasted these differences to zero. While we observed numerical differences in the levels of fits in a few time-frequency periods (Supplementary Figure 8), these did not survive correction for multiple comparisons (shuffling model labels 1,000 times: all $p > 0.6$). In addition, we correlated the concatenated time-frequency maps of BL-NET fits and category model fits in each participant independently. We observed a mean correlation of 0.635 ± 0.035 (Mean ± STD), and R2 of 0.4 ± 0.04 (Mean ± STD), corresponding to the shared variance of the time frequency maps (Supplementary Figure 8). These results suggest that categorical information is an important dimension of representation during the prioritization period in the PFC. We note however that in this comparison, the fits of the category model and the BL-NET are performed on the same neural data, and therefore some shared explained variance in the time-frequency maps is expected.

How can these results be reconciled with the fact that categorical information is not sufficient to explain PFC representations during the prioritization period? We tested two hypotheses: First, it may be that a noisy version of the category model would provide a better fit with PFC representations than a version without noise; second, the specific representational geometry of individual exemplars in the BL-NET matches the geometry in the PFC consistently across our group of subjects.

We tested the first hypothesis by simulation analyses. We correlated the category model under different levels of noise with BL-NET representations. We added Gaussian noise to the category model with a mean of 0 and standard deviations of 0, 0.1, 0.5, 1, 3, 5, and 10. We computed the correlation of the resulting noise models with BL-NET representations (layer 7) 1,000 times (Supplementary Figure 9A). We found that BL-NET representations were significantly more similar to PFC representations during the prioritization period than they were to the noisy version of the category model representations. The correlations of BL-NET and PFC representations resulted in a t-value of 5.88 at the group level (red line in histogram plots, Supplementary Figure 9A). Adding noise to the category model never reached a similar level, in any of the permutations and the tested levels of noise. Notably, results in this simulation demonstrate that the BLNET RSM is not a noisy version of the category model, because adding

random noise to the category model never leads to a similar level of fit with the neural data as the BL-NET model.

To test the second hypothesis, we investigated how consistently the BL-NET reflects the representational geometry of stimuli in PFC. If BL-NET is a good model of PFC representations, we would expect to see that the representational geometries are closely related in most of our subjects. We thus computed the correlation of neural and BL-NET RSMs during the prioritization period in each subject independently. We focused on the cluster of significant time-frequency bins in the original BL-NET analysis (layer seven, beta range). We also computed a distribution of correlation values expected by chance for every subject, by shuffling the labels of the model RSMs 1,000 times. Results are plotted in figure Supplementary Figure 9B, which indicates the percentage of times in each subject that the real correlations with BL-NET were higher than the correlations expected under the null hypothesis. Notably, the fit was > 88% in 11 out of 15 subjects. The fact that we observed this consistent representational geometry in our group of subjects makes it highly unlikely that the that the group level correlation we observed in the main analysis is driven just by noise. These additional analyses demonstrate that while the geometry of representations in PFC is categorically structured, and thus could be considered a ‘variant of category-specificity’, the fine-grained structure of between-category similarities cannot be captured by noise, but by the very specific representational geometry of the BL-NET model.

Supplementary Note 6: Isolating the effect of recurrency

The DNNs employed in our study differ in several aspects, including the number and type of layers, trainable parameters, information processing dynamics, etc. For example, BL-NET employs convolutional, batch normalization, and ReLU layers, while AlexNet incorporates additional layers like dropout, max pooling, and fully connected layers. Furthermore, CorNet-RT exhibits distinct information propagation across layers compared to BL-NET due to the absence of inter-layer information flow before information has been processed in each individual layer. Despite these variations, it is crucial to recognize the shared aspects of these DNNs. All three are convolutional DNNs that learn visual filters in a hierarchical manner across layers. They share the same objective function (classification), learning algorithm (backpropagation), and training data (ImageNet). Our choice of AlexNet as the feedforward model was due to its widespread adoption in cognitive neuroscience research, particularly within the domain of iEEG, which allows for direct comparison with prior studies (e.g., refs. [7,8]). Regarding our recurrent models, we opted for convolutional recurrent networks that are widely known in the cognitive and computational neuroscience community and have been employed to explain important features of visual perception (see refs. [15,16]).

To control potentially confounding factors when isolating the specific effect of recurrence, we employed three pretrained parameter-matched variants of BL-NET, all lacking lateral recurrence but including additional parameters (see ref. [15]): BK-NET, which increases the size of the convolutional kernels; BF-NET, which increases the number of feature maps; and BD-NET, which increases the depth of the network (number of layers). Additionally, for completeness, we included the non-recurrent version of CorNet-RT, named CorNet-Z, which contains a different number of parameters but maintains the number of layers of CorNet-RT.

Focusing on representations in the respective last layer in each of these control architectures (layer 7 in BK-NET and BF-NET, layer 13 in BD-NET, and layer 4 in CorNet-Z), we did not observe any significant fit with representations in non-recurrent networks in the PFC (BK-NET all $p_{corr} > 0.196$; BF-NET, all $p_{corr} > 0.063$; BD-NET, all $p_{corr} > 0.21$; CorNet-Z all $p_{corr} > 0.9$). In the VVS, on the other hand, we did observe a significant fit in BF-NET layer 4 ($p_{corr} = 0.042$), while all other layers did not show significant fits (all $p_{corr} > 0.056$). This was observed in a time period from 2.1s to 2.9s, overlapping in time with the clusters observed in layers 4, 5 and 6 of the original BL-NET analysis. The other control models did not show significant fits to VVS representations in any layer (all $p_{corr} > 0.066$). The CorNet-Z model did not show a significant fit in any layer (all $p_{corr} > 0.23$).

These control analyses, together with the main findings reported in our study (significant fits in PFC locked to the retro-cue for both BL-NET and CorNet-RT), suggests that recurrent computations play an important role in information processing dynamics in the PFC. On the other hand, they also suggest that VVS representations during maintenance can be explained by computations in feedforward models as well.

Supplementary Note 7: Analysis with matched numbers of participants

An important objective of our manuscript is to characterize how representations in PFC and VVS differ during VWM maintenance and prioritization, and for this purpose it is crucial to consider the differences in sample size and, as a result, statistical power in our two ROIs. Focusing on the EMS analysis, we systematically matched the number of subjects in the two regions by randomly removing a total of 11 subjects in the VVS analysis 1,000 times in order to assess the stability of the effect in the VVS under similar levels of statistical power. This led to a total of N=15 subjects in the VVS analysis (note that one subject with only one electrode had already been excluded in the PFC analysis). We focused on the theta band where the most prominent EMS effects were observed in the original analysis. To avoid statistical noise and random fluctuations, we took the average EMS difference (same minus different categories) in the time period of 1.5s after cue offset during maintenance and for all time points during encoding (where most of the effects were observed in the analysis including all subjects) at every random subsampling of subjects. We then contrasted this average difference value against zero at the group level to assess statistical significance. These analyses confirmed significant category-level EMS effects in the VVS with participant numbers matched to those in the PFC in 95.8% of the random subsamples (Supplementary Figure 11A).

In addition to the EMS analysis, we also correlated representations in neural and model RSMs (BL-NET analysis) during encoding and maintenance by randomly subsampling from the total number of subjects in the VVS 1,000 times. We specifically focused on the levels of fits in the last layer of the network during encoding and maintenance. During encoding, we selected the time-frequency cluster observed in layer 6, which covers the whole time period of image presentation (0-800ms), and frequencies in the 3-150Hz range. We took the average levels of fit in this cluster and contrasted them against zero at the group level. Results revealed that the average fits with the BL-NET during the encoding period was significant in 100% of the random VVS subsamples (Supplementary Figure 11B). During maintenance, we specifically focused on the cluster observed in layer 6 of BL-NET in the VVS analysis, and again took the average levels of fit in this cluster at every subsample. Results revealed that fits with maintenance were significant in this analysis in 100% of the random subsamples (Supplementary Figure 11C).

Supplementary References

1. Baek, S., Song, M., Jang, J., Kim, G. & Paik, S.-B. Face detection in untrained deep neural networks. *Nat. Commun.* **12**, 7328 (2021).
2. Bao, P., She, L., McGill, M. & Tsao, D. Y. A map of object space in primate inferotemporal cortex. *Nature* **583**, 103–108 (2020).
3. Cadieu, C. F. *et al.* Deep neural networks rival the representation of primate IT cortex for core visual object recognition. *PLoS Comput. Biol.* **10**, e1003963 (2014).
4. Cichy, R. M., Khosla, A., Pantazis, D., Torralba, A. & Oliva, A. Comparison of deep neural networks to spatio-temporal cortical dynamics of human visual object recognition reveals hierarchical correspondence. *Sci. Rep.* **6**, 27755 (2016).
5. Khaligh-Razavi, S.-M. & Kriegeskorte, N. Deep supervised, but not unsupervised, models may explain IT cortical representation. *PLoS Comput. Biol.* **10**, e1003915 (2014).
6. Kuzovkin, I. *et al.* Activations of deep convolutional neural networks are aligned with gamma band activity of human visual cortex. *Commun. Biol.* **1**, 1–12 (2018).
7. Liu, J. *et al.* Stable maintenance of multiple representational formats in human visual short-term memory. *Proc. Natl. Acad. Sci.* **117**, 32329–32339 (2020).
8. Liu, J. *et al.* Transformative neural representations support long-term episodic memory. *Sci. Adv.* **7**, eabg9715 (2021).
9. Tang, H. *et al.* Recurrent computations for visual pattern completion. *Proc. Natl. Acad. Sci.* **115**, 8835–8840 (2018).
10. Vinken, K. & Op de Beeck, H. Using deep neural networks to evaluate object vision tasks in rats. *PLOS Comput. Biol.* **17**, e1008714 (2021).
11. Mehrer, J., Spoerer, C. J., Jones, E. C., Kriegeskorte, N. & Kietzmann, T. C. An ecologically motivated image dataset for deep learning yields better models of human vision. *Proc. Natl. Acad. Sci.* **118**, e2011417118 (2021).
12. Everling, S., Tinsley, C. J., Gaffan, D. & Duncan, J. Filtering of neural signals by focused attention in the monkey prefrontal cortex. *Nat. Neurosci.* **5**, 671–676 (2002).
13. Wallis, J. D., Anderson, K. C. & Miller, E. K. Single neurons in prefrontal cortex encode abstract rules. *Nature* **411**, 953–956 (2001).
14. Cromer, J. A., Roy, J. E. & Miller, E. K. Representation of Multiple, Independent Categories in the Primate Prefrontal Cortex. *Neuron* **66**, 796–807 (2010).
15. Spoerer, C. J., Kietzmann, T. C., Mehrer, J., Charest, I. & Kriegeskorte, N. Recurrent neural networks can explain flexible trading of speed and accuracy in biological vision. *PLoS Comput. Biol.* **16**, e1008215 (2020).
16. Kubilius, J. *et al.* Cornet: Modeling the neural mechanisms of core object recognition. *BioRxiv* 408385 (2018).
