# Supplementary material for: Maintenance and transformation of representational formats during working memory prioritization
Source: Nat Commun. 2024 Sep 19;15:8234. doi: 10.1038/s41467-024-52541-w (PMC11412997; doi:10.1038/s41467-024-52541-w)
Supplement: Supplementary file 3 — Reporting Summary [file 41467_2024_52541_MOESM3_ESM.pdf]

nature portfolio

Corresponding author(s): Daniel Pacheco

Last updated by author(s): Oct 5, 2023

Reporting Summary

Nature Portfolio wishes to improve the reproducibility of the work that we publish. This form provides structure for consistency and transparency in reporting. For further information on Nature Portfolio policies, see our Editorial Policies and the Editorial Policy Checklist.

Statistics

For all statistical analyses, confirm that the following items are present in the figure legend, table legend, main text, or Methods section.

n/a	Confirmed	
☐	☒	The exact sample size (*n*) for each experimental group/condition, given as a discrete number and unit of measurement
☐	☒	A statement on whether measurements were taken from distinct samples or whether the same sample was measured repeatedly
☐	☒	The statistical test(s) used AND whether they are one- or two-sided<br>*Only common tests should be described solely by name; describe more complex techniques in the Methods section.*
☐	☒	A description of all covariates tested
☐	☒	A description of any assumptions or corrections, such as tests of normality and adjustment for multiple comparisons
☐	☒	A full description of the statistical parameters including central tendency (e.g. means) or other basic estimates (e.g. regression coefficient) AND variation (e.g. standard deviation) or associated estimates of uncertainty (e.g. confidence intervals)
☐	☒	For null hypothesis testing, the test statistic (e.g. *F*, *t*, *r*) with confidence intervals, effect sizes, degrees of freedom and *P* value noted<br>*Give P values as exact values whenever suitable.*
☒	☐	For Bayesian analysis, information on the choice of priors and Markov chain Monte Carlo settings
☒	☐	For hierarchical and complex designs, identification of the appropriate level for tests and full reporting of outcomes
☐	☒	Estimates of effect sizes (e.g. Cohen's *d*, Pearson's *r*), indicating how they were calculated

Our web collection on statistics for biologists contains articles on many of the points above.

Software and code

Policy information about availability of computer code

Data collection	The experiment was programmed in Presentation (Neurobehavioral systems, California, USA), and was deployed on Samsung 12'' tablet computers running Microsoft Windows.
Data analysis	Data was analyzed using custom written code in Matlab and Python. Several Matlab toolboxes were employed for the iEEG data analysis, including EEGLAB and Fieldtrip. The AlexNet model was taken from the Deep Learning Toolbox (Matlab). Recurrent neural networks models were obtained from https://github.com/cjspoerer/rcnn-sat (BL-NET) and https://github.com/dicarlolab/CORnet (CORnet-RT)

For manuscripts utilizing custom algorithms or software that are central to the research but not yet described in published literature, software must be made available to editors and reviewers. We strongly encourage code deposition in a community repository (e.g. GitHub). See the Nature Portfolio guidelines for submitting code & software for further information.

Data

Policy information about availability of data

All manuscripts must include a data availability statement. This statement should provide the following information, where applicable:

- Accession codes, unique identifiers, or web links for publicly available datasets
- A description of any restrictions on data availability
- For clinical datasets or third party data, please ensure that the statement adheres to our policy

Anonymized intracranial EEG data and custom-written Python and Matlab code supporting the findings of this study will be made available upon publication through the Open Science Framework.

Research involving human participants, their data, or biological material

Policy information about studies with human participants or human data. See also policy information about sex, gender (identity/presentation), and sexual orientation and race, ethnicity and racism.

Reporting on sex and gender	We collected data from both sexes in this study (self-reported), and did not evaluated differences related to sex for performing the task.
Reporting on race, ethnicity, or other socially relevant groupings	No distinctions were made regarding race or ethnicity. The experiment was conducted with different patient populations in Germany and China, and data from the two patient populations was aggregated in all analyses.
Population characteristics	Thirty-two patients (17 females, 30 ± 10.04 years) with medically intractable epilepsy participated in the study.
Recruitment	Data were collected at the Freiburg Epilepsy Center, Freiburg, Germany; the Epilepsy center, Second Affiliated Hospital, School of Medicine, Zhejiang University, Hangzhou, China; and the Center of Epileptology, Xuanwu Hospital, Capital Medical University, Beijing, China. All patients were implanted for clinical purposes and participation in the study was voluntary.
Ethics oversight	The study was conducted according to the latest version of the Declaration of Helsinki and approved by the responsible ethics committees at the hospitals in Freiburg, Hangzhou and Beijing. All patients provided written informed consent.

Note that full information on the approval of the study protocol must also be provided in the manuscript.

Field-specific reporting

Please select the one below that is the best fit for your research. If you are not sure, read the appropriate sections before making your selection.

[x] Life sciences   [ ] Behavioural & social sciences   [ ] Ecological, evolutionary & environmental sciences

For a reference copy of the document with all sections, see nature.com/documents/nr-reporting-summary-flat.pdf

Life sciences study design

All studies must disclose on these points even when the disclosure is negative.

Sample size	We determined the sample size based on previous iEEG studies. We aimed to obtain at least 15 subjects with electrodes in the Prefrontal Cortex (PFC) and the Ventral Visual Stream (VVS). Our sample size of N=32 patients is higher than similar iEEG studies in the field.
Data exclusions	Data from patients with only 1 electrode in specific brain regions were excluded as indicated in the manuscript (Methods). One participant out of 16 with PFC electrodes was excluded of all PFC analysis. Two participants with only 1 electrode in the VVS (out of 30) were excluded from all VVS analyses.
Replication	We applied different neural network architectures to verify the main findings of our study (two recurrent models), and performed the analysis using variants of DNNs trained with two different image sets.
Randomization	All participants took part in all experimental conditions in our study following a within subjects design.
Blinding	Investigators were blind to experimental conditions during data collection and during the preprocessing of the data (artifact rejection) as explained in the manuscript (Methods section).

Reporting for specific materials, systems and methods

We require information from authors about some types of materials, experimental systems and methods used in many studies. Here, indicate whether each material, system or method listed is relevant to your study. If you are not sure if a list item applies to your research, read the appropriate section before selecting a response.

Materials & experimental systems

n/a	Involved in the study
☒	☐ Antibodies
☒	☐ Eukaryotic cell lines
☒	☐ Palaeontology and archaeology
☒	☐ Animals and other organisms
☒	☐ Clinical data
☒	☐ Dual use research of concern
☒	☐ Plants

Methods

n/a	Involved in the study
☒	☐ ChIP-seq
☒	☐ Flow cytometry
☒	☐ MRI-based neuroimaging

Plants

Seed stocks	*Report on the source of all seed stocks or other plant material used. If applicable, state the seed stock centre and catalogue number. If plant specimens were collected from the field, describe the collection location, date and sampling procedures.*
Novel plant genotypes	*Describe the methods by which all novel plant genotypes were produced. This includes those generated by transgenic approaches, gene editing, chemical/radiation-based mutagenesis and hybridization. For transgenic lines, describe the transformation method, the number of independent lines analyzed and the generation upon which experiments were performed. For gene-edited lines, describe the editor used, the endogenous sequence targeted for editing, the targeting guide RNA sequence (if applicable) and how the editor was applied.*
Authentication	*Describe any authentication procedures for each seed stock used or novel genotype generated. Describe any experiments used to assess the effect of a mutation and, where applicable, how potential secondary effects (e.g. second site T-DNA insertions, mosiacism, off-target gene editing) were examined.*
